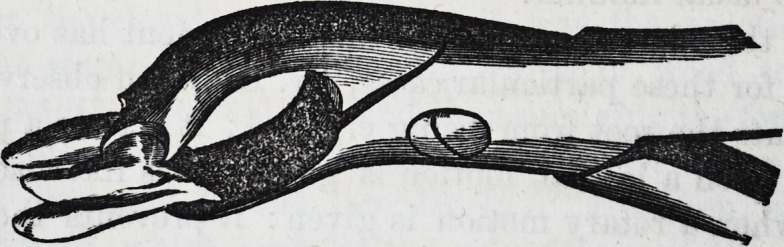# The Adapted Forceps

**Published:** 1858-01

**Authors:** J. Robinson


### ARTICLE XIV.

*The Adapted Forceps.* By J. Robinson, Esq.

For the removal of the central and lateral deciduous teeth in the lower jaw, one pair of forceps only is required—a narrow-beaked hawk's-bill forceps. Thus—

For the canines in the lower jaw an instrument of this decription—middle size hawk's-bill is best adapted.

For the removal of the temporary molars in the lower jaw, only one pair of forceps is necessary.

Now for the extraction of the permanent teeth the forceps should be much larger, wider in the grip, and of stronger manufacture. It is desirable to have the blades fastened with a single screw, admitting of slight looseness or play between them, so as more redily to fit the various sizes of the teeth. For the centrals and canines one pair only is necessary.

A smaller pair for the laterals. Thus—

One pair only is used for the removal of the bicuspids, which may either be slightly curved or straight, but narrower in the inner than in the outer groove of the blade.

For the first and second molars, the instrument ought to be curved. Some operators, however, prefer curved for the second, and straight for the first molars; in either, the outer blade must have two grooves, in the centre of which extends a spine three or four lines above the grooves, in order that when the instrument is applied, this spine may adapt itself between the two external roots. The inner blade should be grooved, with rounded corners, to fit the palatine neck of the tooth.

It is well for the operator to be provided with a pair of a smaller size of the same description, and coming closer together at the beaks or points of contact, as the sizes of these teeth may vary, and he might find that in attempting to operate with the larger size, he has no grip upon the neck of the tooth, when if the operation were proceeded with, a fracture and a separation of the crown from the fangs would be the inevitable result. In the subjoined cut of the instrument, a curvature takes place from the joint, and extends so far, that in operating it does not injure the lower teeth on the left side of the patient; the handle terminates in a hook, which is intended to pass round the little finger, and gives the operator greater power while in the act of performing the several movements necessary for the extraction of a tooth. Some practitioners, who possess the facility of using the left hand as well as the right, prefer the hook on the right instead of the left side of the forceps.

The *dentes sapientiæ*, or wisdom teeth, of the upper jaw require for their extraction an instrument bent above the joint, so as to form two right angles (as represented at bottom of page 69,) which enables the operator to see distinctly the tooth whilst operating.

However expert and judicious an operator may be in the use of extracting instruments, he will occasionally find that a portion of the external wall of the alveolar process has been fractured. In this case, it is better to remove the fractured portion than to allow it to remain in the mouth of the patient to go through the process of exfoliation; and impress him and his friends with the idea that a fractured jaw has been the result of the operation. Any loose portions of the alveolar process may, immediately after extraction, be removed with a pair of bone-forceps. In my own practice, when preparing a mouth for artificial teeth, I make it a rule, while the patient is under the influence of chloroform, or otherwise, to remove all the edges and points by the aid of bone nippers. The following engraving represents forceps for the removal of *spicula* of the alveolar process from the upper and lower jaws.

The subjoined excising forceps are used for clipping the edges of the alveolar process—the straight for the anterior, and the curved for the posterior part of the mouth.

For the lower jaw it will be necessary to have the forceps curved at various angles, and for the removal of the incisors, cuspidati and bicuspids, hawk's bills of various sizes and angles are essential.

Occasionally, when a bicuspid has been developed within the dental circle, obstructing the movement of the tongue, it will be impracticable to remove the irregular tooth by means of any of the above instruments without danger either to the adjoining teeth, or fracture of the one to be extracted. For this purpose I have constructed the following instruments, which are applicable to either jaw ; the smaller beak being intended to fit between the teeth.

In removing the *dentes sapientœ* in the lower jaw, the forceps should be somewhat curved from the joint, like the former, but not bent to so great an angle as those used for the molars. Those are to be preferred that have a slightly rounded point in the centre of the grooved blades; thus—

The first and second molars are extracted by means of forceps, the outer beak of which is considerably more curved than the inner, both closing in a point so as to reach the bifurcation of the fangs, and so bent from the joints that they clear the upper jaw. This cut represents the instrument with curved handles for the right side of the lower jaw. Some operators prefer instruments with straight handles, and bent at various angles.

The following engraving represents one of those accidents which occasionally occur, in consequence of the careless employment of the forceps, in which, either from using too large an instrument, and taking hold of the alveolar process, or from embracing two of the teeth at the same time, a considerable piece of the alveolar process has been torn away.

For the removal of single stumps in either jaw, the forceps should be well grooved, and tapering to one edge, until within a few lines at their points, to allow of their being pressed down between the gums and the fangs of the tooth. Those used for the upper jaw should be straight or curved, as in the subjoined cut.

For the lower jaw the forceps should be curved, some at right angles, and the others nearly so.

In the case of stumps deeply seated in the back of the jaw, whether such stumps have been the result of disease, or remain after the improper or unsuccessful use of the key or forceps, admirable instruments called elevators have been invented—they should be slightly curved, strong in the blade, and well tempered to prevent bending or breaking. The first of the following engravings represents one employed for the back of the lower jaw, the other for the sides of the upper jaw. However, where the stump forceps can be used for the removal of roots, they should be preferred.

The six front teeth in the upper jaw are sometimes so completely hollowed out by caries, as to render the success of an operation for their removal extremely doubtful, on account of the thinness of the outer walls, by which they are rendered incapable of bearing the pressure of either the forceps or the elevator. Formerly, it was the practice to render the stump solid, by shaping a piece of wood to the size and length of the hollow, and then applying the forceps. For these otherwise forlorn cases, an admirable instrument has been invented by the late Dr. S. P. Hullihen, of the United States, which contains the advantages of the screw and forceps. In describing this ingenious invention, the doctor says: "Lengthwise, within and between the blades of the beak, is a steel tube, one end of which is open, the other solid and flat, and jointed in a mortice in the small part of the joint of the forceps. When the forceps are opened, the joint permits the tube to fall backwards and forwards from one blade of the beak to the other, with lateral motion. Within this tube is a spiral spring, which forces up a shaft two-thirds of the tube; the other part is a well-tapered conical screw. The screw and tube are so fitted together to the beak of the forceps, that one-half of the rounded part of the shaft projects beyond the end of the tube, so that the shaft may play up and down upon the spring about half an inch, and the screw or shaft be embraced between the blades of the beak of the instrument."

The manner of using this instrument is by first grasping the upper extremity of the screw between the blades of the forceps, and gently turning the handle of the instrument so as to drive the screw into the root of the tooth as far up as possible. The blades of the forceps are now opened, and pushed forward on the fang, which is grasped and extracted in the usual manner.

On the advantages which this instrument has over every other for these particular cases, Dr. Hullihen observes: "It prevents the root from being crushed; it acts as a powerful lever when a lateral motion is given: it is likewise of service when a rotary motion is given: it prevents the forceps from slipping, or the action being lost, should even one side of the root give way in the act of extracting it; and it is used with equal advantage when one root is entire gone."

In my practice, I have had many opportunities of testing the merits of this invention, and I can safely affirm that it is one of the most valuable auxiliaries to the scientific practice of dental surgery that has ever been introduced.

Dr. W. H. Elliot, an able practitioner, has also invented a clever instrument for removing fangs of teeth by means of a screw, which is inserted into an universal joint upon the end of the instrument. This instrument may, it is said, be applied with equal facility to the fangs of the molars, or to those of the front teeth.

For the removal of bicuspid stumps in the upper jaw, we have found the straight narrow-beaked forceps used for the deciduous centrals best adapted.

Caries will frequently extend and destroy the entire crown of the upper molars down to the edge of the gum. To meet these cases, I have constructed two pairs of forceps, for right and left sides. In these forceps, instead of the point of the outer blade terminating a few lines beyond the grooves, as in ordinary molar forceps, it is carried forwards and inwards, ending in a cutting-edge point, a few lines down this projecting spine, the object of this being to cut the alveolus between the external fangs, high up, whilst the inner blade rests upon the alveolus, and embraces the palatine fang.

The operator, by this instrument, obtains a firm grasp, and is enabled to remove these stumps without difficulty, when separated by caries.

Dr. Maynard, of America, has also invented an instrument for the same purpose, on which, instead of the ordinary blade, a kind of conical hook, terminating in a point, is used for perforating the alveolus between the outer fangs. I, however, prefer my own in practice, as it gives the operator a firm hold, by means of the grooves in the blades around the outer fangs, while in Dr. Maynard's, the points of contact are only between and at their bifurcation.

In describing the manner in which the instrument for the extraction of teeth, in this paper, should be used, I may here observe, that my instructions are given to pupils, not to the *initiated;* that the mechanical motions are very similar for all the teeth.

I shall, therefore, select a pair of straight forceps for the removal of the *incisors* in the *upper jaw*, taking care that the instrument is sufficiently wide in the beak to clear the crown, but only so wide as to embrace the neck of the tooth, and as far as the alveolar process, or a serious loss of that bone may result from the operation. If the crown only be grasped, the tooth will, in all probability, be crushed by the pressure employed. Having grasped the tooth, alternate lateral, with slight rotary motions, are given in quick succession, until the tooth yields, when a slight downward action will easily remove it.

Provided the tooth be much decayed and hollowed out in the centre, the pupil, if he uses the straight forceps, should run the point well up under the gum, and employ only sufficient pressure to prevent the instrument from slipping: or if he is provided with a pair of screw forceps, he can frequently use them with advantage in these cases.

Presuming the pupil is not in possession of a pair of Hullihen's screw forceps, he should have recourse to the wooden plug previously referred to. An occasional failure in these cases must be expected, and the pupil must not be disheartened, for the very best dental practitioners have occasionally failed in extracting the fang of a tooth under similar circumstances. For the removal of the cuspidatus in the upper jaw, the same alternate lateral and downward motions are necessary, but with a *much less* rotary action. These teeth usually require a much greater amount of physical force in combination with the mechanical to remove them from their sockets; and occasionally are flattened, and curved at the apex of their fangs; and this portion is very liable to be fractured, and left in the sockets. To extract the remaining portion is a very difficult operation, and can only be effected by cutting through the external wall of the alveolar process, and removing the fractured part, either by a small elevator, or finely-pointed stump forceps.

The bicuspids in ordinary cases are readily extracted, and require merely the alternate and lateral movements, tending to the perpendicular. In those where caries has undermined and destroyed the greater part of the dentine of the crown, leaving in many cases nothing but mere walls of enamel, the points of the forceps should be forced well up to the edge of the alveolar process.

In other cases again, in these teeth caries will frequently undermine the enamel, and continue its course a considerable distance up the centre of the fang, and from some mechanical injury the whole crown is suddenly broken down, or in others only half the crown may be fractured. In either case the ordinary application of the forceps would render the remaining portion liable to fracture. To make the operation of extraction more certain in such instances, it is necessary to separate the gum freely with a lancet, both externally and internally; the beaks of the forceps should be run high up, and embrace both edges of the alveolar process. A firm and decided pressure should now be made, so as to grip the fang, which should be instantly followed by the usual mechanical movements. After extraction, the pupil, upon examination, will frequently find small pieces of loose alveolar process remain; these should be removed with his bone forceps; he will also find in some of the more difficult of those cases, that exfoliation of small portions of the process, will continue to annoy the patient for some weeks, which may temporarily alarm him; the frequent application of the spongeopiline, steeped in hot water or milk, or the application of a leech to the part, with a dose or two of some mild aperient, will generally be sufficient to reduce all local inflammation, and the loose portions of bone will be exfoliated.—*London Quarterly Jour. Dent. Sci.*

## Figures and Tables

**Figure f1:**
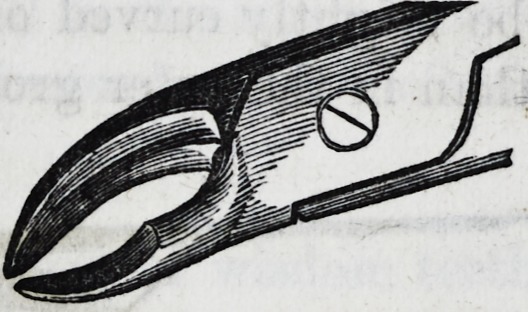


**Figure f2:**
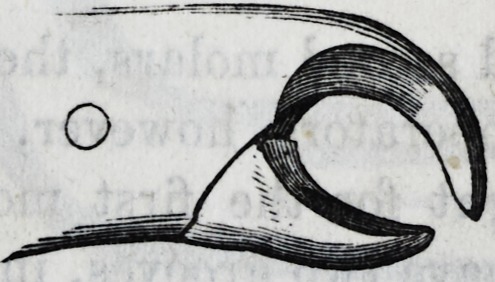


**Figure f3:**
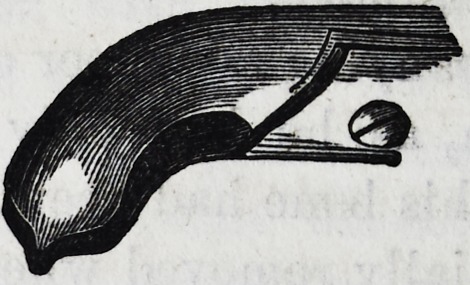


**Figure f4:**
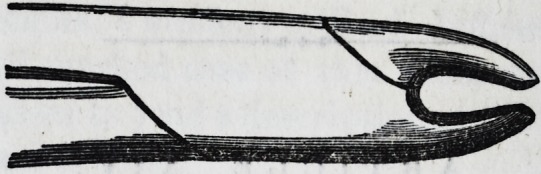


**Figure f5:**
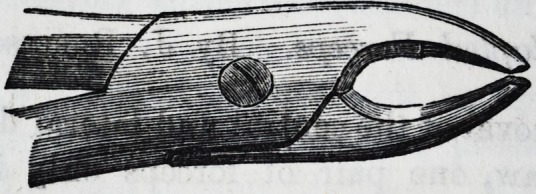


**Figure f6:**
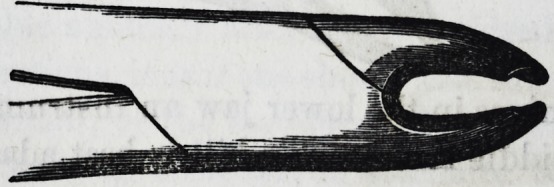


**Figure f7:**
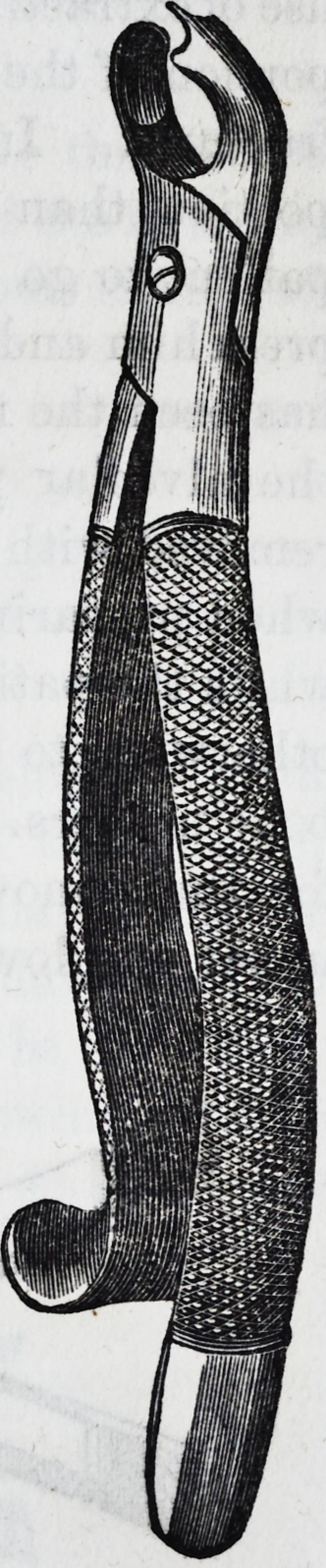


**Figure f8:**
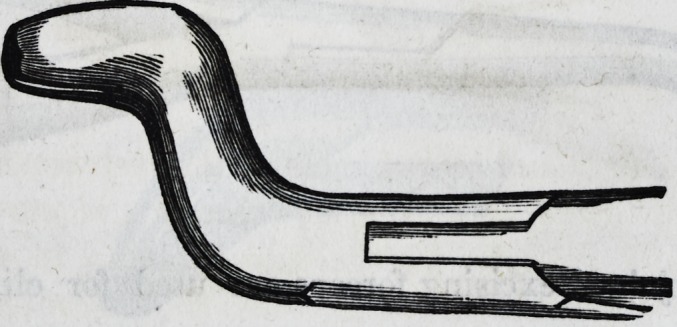


**Figure f9:**
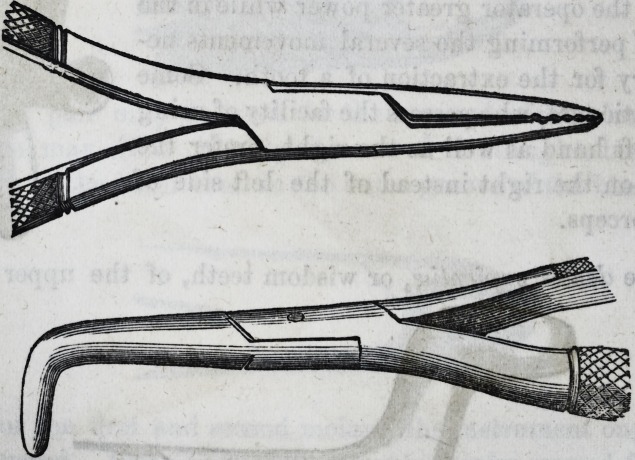


**Figure f10:**
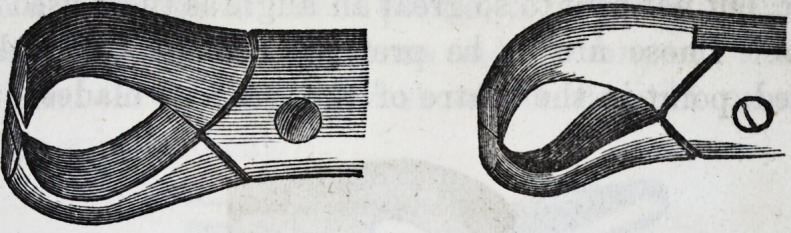


**Figure f11:**
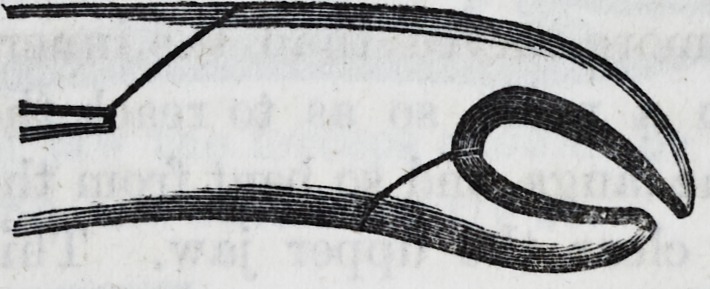


**Figure f12:**
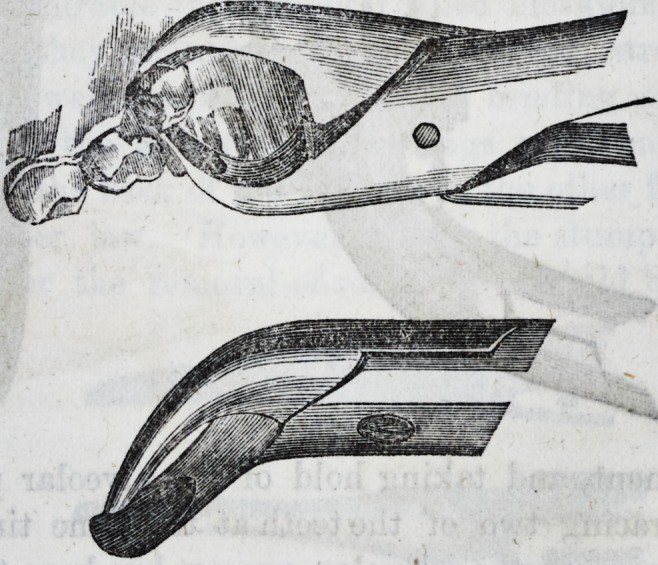


**Figure f13:**
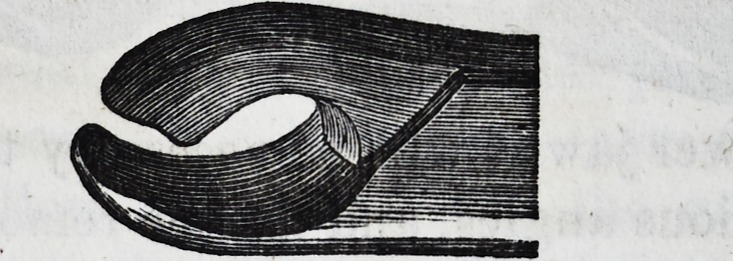


**Figure f14:**
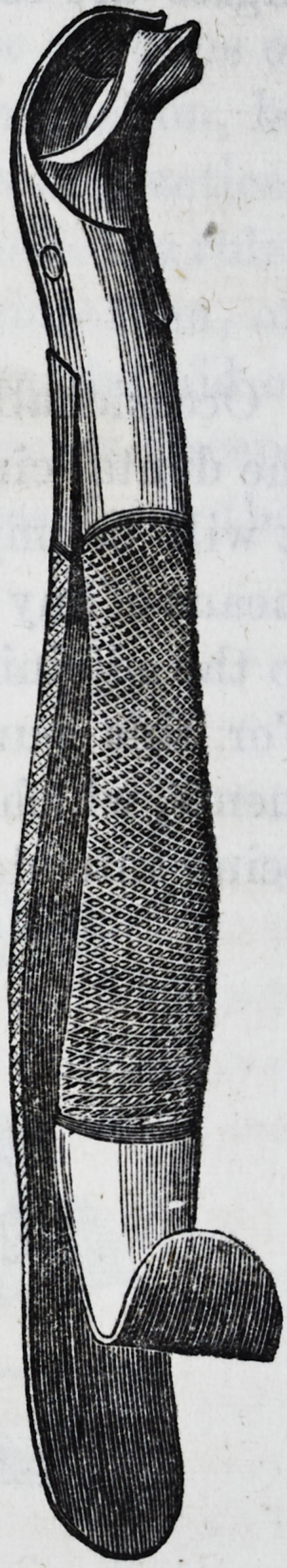


**Figure f15:**
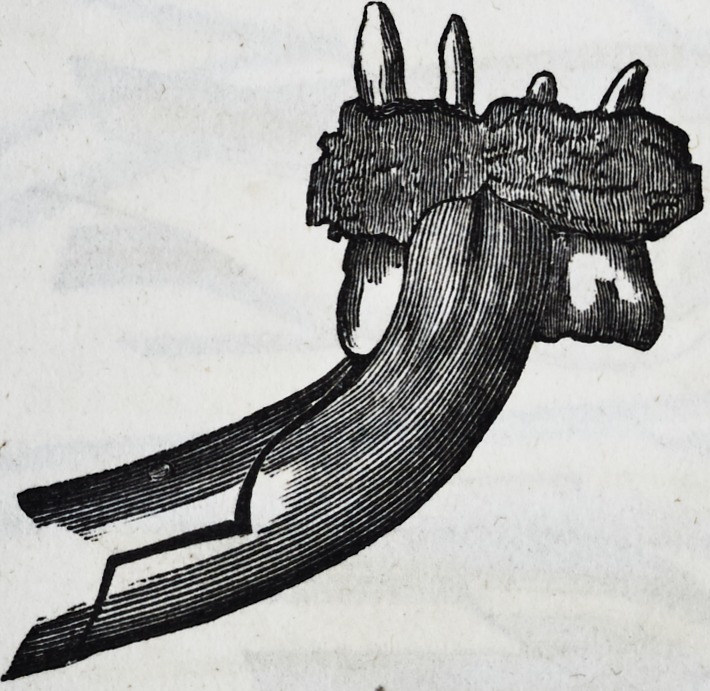


**Figure f16:**
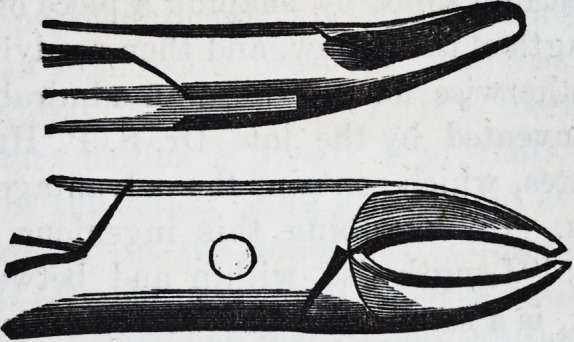


**Figure f17:**
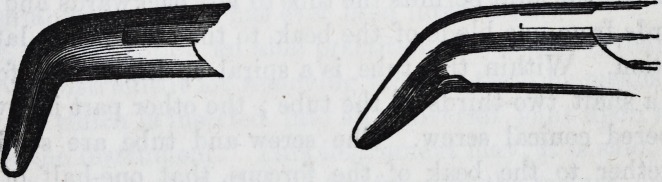


**Figure f18:**
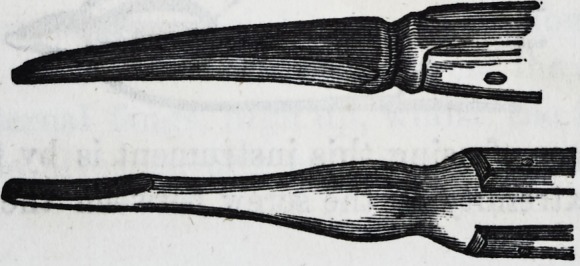


**Figure f19:**
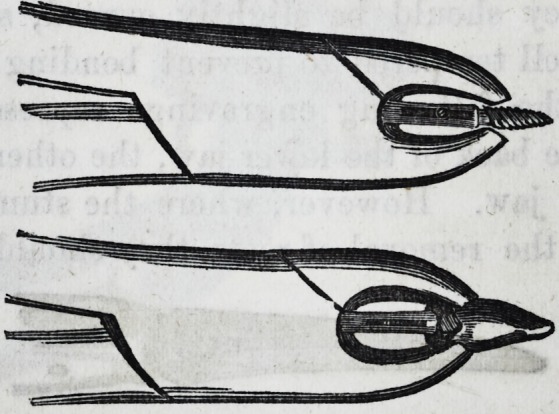


**Figure f20:**
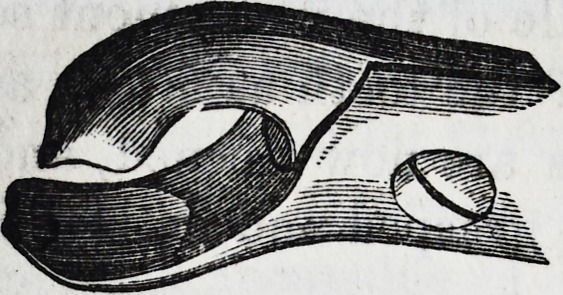


**Figure f21:**